# Post-transcriptional control by RNA-binding proteins in diabetes and its related complications

**DOI:** 10.3389/fphys.2022.953880

**Published:** 2022-10-06

**Authors:** Shiyu Zhang, Xiaohua Yang, Miao Jiang, Lianhua Ma, Ji Hu, Hong-Hong Zhang

**Affiliations:** ^1^ Department of Endocrinology, The Second Affiliated Hospital, Soochow University, Suzhou, China; ^2^ The Affiliated Haian Hospital of Nantong University, Nantong, China

**Keywords:** RNA-binding proteins, RNA-protein interaction, chronic complications, post-transcriptional gene regulation, diabetes mellitus

## Abstract

Diabetes mellitus (DM) is a fast-growing chronic metabolic disorder that leads to significant health, social, and economic problems worldwide. Chronic hyperglycemia caused by DM leads to multiple devastating complications, including macrovascular complications and microvascular complications, such as diabetic cardiovascular disease, diabetic nephropathy, diabetic neuropathy, and diabetic retinopathy. Numerous studies provide growing evidence that aberrant expression of and mutations in RNA-binding proteins (RBPs) genes are linked to the pathogenesis of diabetes and associated complications. RBPs are involved in RNA processing and metabolism by directing a variety of post-transcriptional events, such as alternative splicing, stability, localization, and translation, all of which have a significant impact on RNA fate, altering their function. Here, we purposed to summarize the current progression and underlying regulatory mechanisms of RBPs in the progression of diabetes and its complications. We expected that this review will open the door for RBPs and their RNA networks as novel therapeutic targets for diabetes and its related complications.

## 1 Introduction

Diabetes mellitus is one of the fastest-growing metabolic disorders characterized by chronic hyperglycemia. In recent decades, the global prevalence of diabetes in adults has been growing at an astonishing rate. It is estimated that in 2045 there will be 693 million adults who suffered from diabetes worldwide (Cho et al., 2018; Harding et al., 2019). Depending on the different mechanisms, diabetes can be divided into two main forms, type 1 diabetes mellitus (T1DM) and type 2 diabetes mellitus (T2DM). T1DM is considered an auto-immune disease defined by islets β-cell damage and absolute lack of insulin. T2DM is often accompanied by relatively insufficient insulin secretion and insulin resistance, preventing insulin from stimulating glucose uptake into target tissues, resulting in elevated blood glucose levels (Atkinson et al., 2014; Petersmann et al., 2019; Weir et al., 2020). Persistent hyperglycemia affects nearly every tissue of the body that causes severe macrovascular and microvascular complications, with retinopathy, cardiomyopathy, nephropathy, neuropathy, and peripheral vascular disease serving as key avenues of morbidity (Cole and Florez, 2020). Currently, a thorough knowledge of the molecular pathophysiology of diabetic complications remains elusive. Emerging evidence supports that the RNA-binding proteins are involved in the occurrence and development of diabetes and its complications (Nutter and Kuyumcu-Martinez, 2018; Salem et al., 2019; Good and Stoffers, 2020; Kelaini et al., 2021). The specific mechanisms will be described in detail in the present review.

RBPs are typically considered as proteins that are responsible for modulating post-transcriptional gene expression in the eukaryotic cells (Gerstberger et al., 2014). Thousands of such RBPs have been discovered and investigated over the years. RBPs can recognize and interact with their target RNAs to form ribonucleoprotein (RNP) complexes which control almost every aspect of post-transcriptional processing of target RNA substrates, including pre-mRNA splicing, translational control, cleavage and polyadenylation, RNA stability, RNA localization, nuclear export, and RNA editing (Van Nostrand et al., 2020). In recent years, numerous RBPs have been demonstrated to be involved in many human diseases, from cardiovascular diseases and endocrine dysfunction to cancer, and neurodegenerative disorders (Pereira et al., 2017; Lujan et al., 2018; Yang et al., 2018; Cao et al., 2021; Kelaini et al., 2021; Klim et al., 2021). It has been shown that post-transcriptional dysregulation is linked to diabetes mellitus, which serves as a reminder that RBPs may be crucial in the pathogenesis of diabetes and its related complications (Nutter and Kuyumcu-Martinez, 2018).

In this review, we will provide a brief overview of the mechanisms by which RBPs exercise their functions. Then, we will explain how these RBPs are dysregulated and their contribution to the pathological process of diabetes mellitus. In addition, we would like to focus on the relationships between RBPs and diabetic complications. We believe that a comprehensive understanding of the RBPs’ role in diabetes and its associated complications may aid in the development of innovative treatments in clinic.

## 2 Roles of RNA-binding proteins

### 2.1 Regulators of mRNA life cycle

The mRNA life cycle is a complex system that includes the process of transforming the newly transcribed mRNA molecules to fully functional mature mRNA transcripts. RBPs play an essential role in this process. Recent studies have revealed some RBPs do not have typical RNA binding domains (RBDs) but are replaced by at least one intrinsically disordered region (IDR) through which they can not only be involved in aggregation of RNPs, but also directly engage in RNA binding (Hentze et al., 2018). However, most RBPs are considered to interact with their target RNAs by a limited set of RBDs, such as the RNA recognition motif (RRM), hnRNP K homology domain (KH), zinc-finger, and DEAD/DEAH box helicase (Van Nostrand et al., 2020). The interaction of RBP-RNA occurs at RBD, which is mainly located within 5′and 3′untranslated regions (UTRs) of RNA, although it can also be found at the intronic and exonic regions. RBPs usually have a series of repeats RBDs that work together to improve specificity and affinity for their target mRNAs. Multiple target mRNAs can have their expression controlled by a single RBP. Multiple RBPs can interact with the same mRNA, playing a role in either cooperation or competition (Pope and Medzhitov, 2018; Van Nostrand et al., 2020).

**FIGURE 1:**
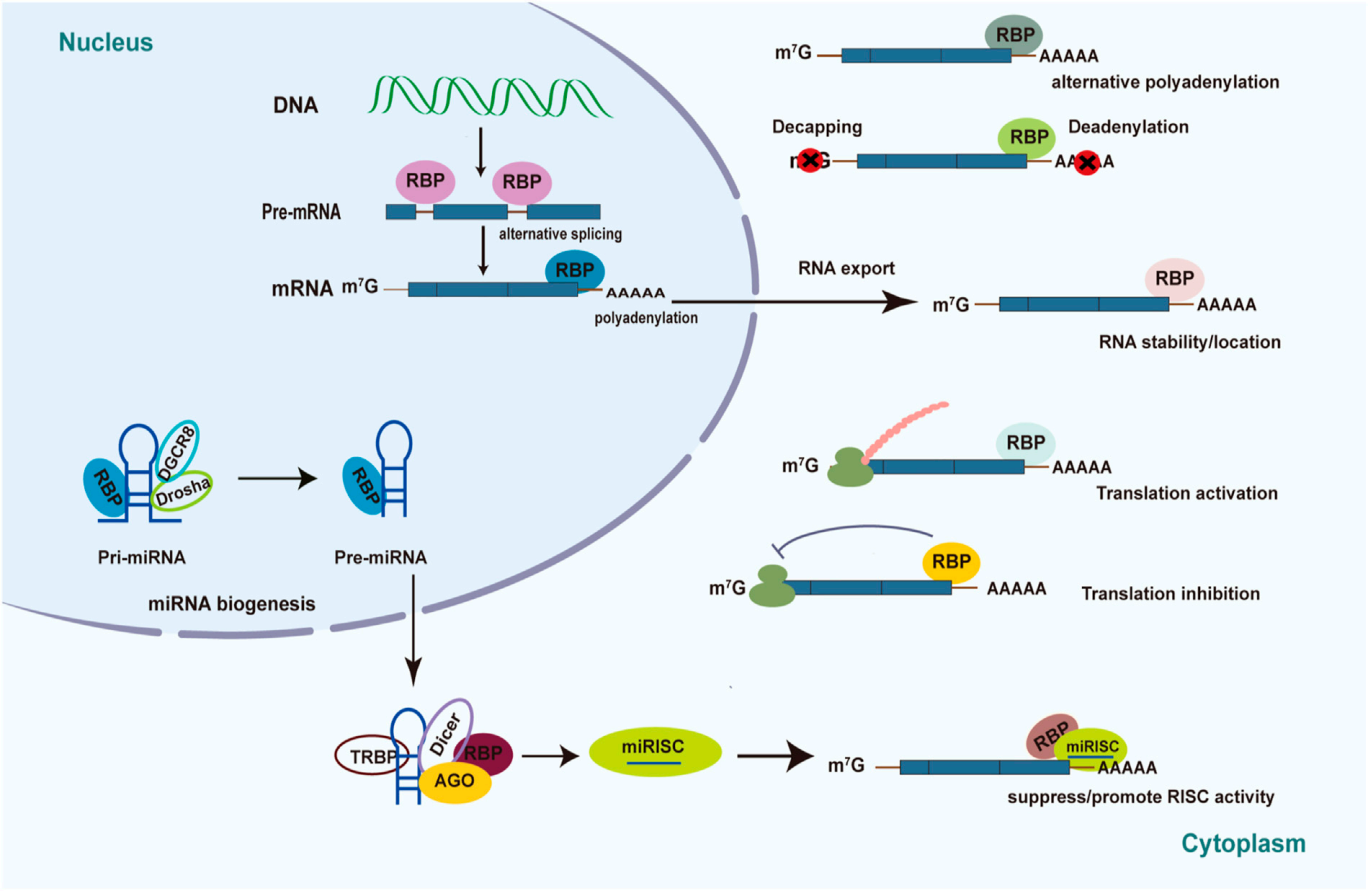
Mechanism of posttranscriptional regulation controlled by RBPs in diabetes. Schematic diagrams summarize the various roles of RBPs played in diabetes pathology and diabetic complications. RBPs have the ability to determine RNA’s fate through pre-mRNA splicing, translational control, polyadenylation, RNA stability, RNA localization, RNA export, and miRNA-mediated processing.

The mechanisms of post-transcriptional regulation by RBPs are complex and elaborate, including 5′capping, alternative splicing of pre-mRNAs, polyadenylation, RNA degradation and stabilization, RNA localization and export, proteins translation (Figure 1) (Corley et al., 2020). RBPs can promote the maturation of RNA *via* pre-mRNA alternative splicing, polyadenylation, RNA editing, and the addition of the 5′ cap in the target RNAs. RBPs can regulate pre-mRNA alternative splicing (AS) through binding to the pre-mRNA and interacting with the spliceosome components, which generate variant protein isoforms from a single gene, resulting in transcriptome and proteome diversity (Sperling, 2017). Previous studies demonstrated that AS controlled by RBPs plays a crucial role in diabetes and its complications (Verma et al., 2013; Nutter et al., 2016; Gazzara et al., 2017; Belanger et al., 2019; Verma et al., 2022). The role of RBPs in AS will be discussed in detail in the next section.

mRNA can be exported from the nucleus to the cytoplasm to perform the function of protein translation. RBPs such as eIF4E is essential for translation of majority of mRNAs. It has been reported that mRNA Cap-binding protein eIF4E can recognize the structure of the 5′-m^7^GTP cap of mRNA and assemble it into eIF4F complexes which can recruit ribosomes onto mRNA to perform translational functions (Lazaris-Karatzas et al., 1990; Osborne and Borden, 2015; Ho and Lee, 2016). However, under diabetic conditions, the formation of eIF4F complexes is inhibited and thus affects the translation rate, contributing to the development of diabetic complications (Schrufer et al., 2010; Dennis et al., 2015; Miller et al., 2016). Besides, RBPs can trigger the degradation and RNA decay process by binding to cis-regulatory RNA elements and recruiting mediators (Pérez-Ortín et al., 2013). For example, 5′ cap can be removed by decapping enzymes while 3′-poly A tail can be diminished by deadenylating enzymes. A well-known element that mediates degradation is the AU-rich element (ARE) located in the 3′ UTRs of mRNA (Mayr, 2019). Multiple studies showed that RBPs such as Tristetraprolin (TTP) and ELAV families can regulate the degradation of target mRNAs that contain the ARE element (Makita et al., 2021; Sidali et al., 2021). Transcription and degradation rates together regulate the content of intracellular mRNA.

### 2.2 Regulators of microRNA life cycle

RBPs can also control the post-transcriptional regulatory process by regulating the biogenesis and function of non-coding RNAs (Ho et al., 2021; Yao et al., 2022). For instance, RBPs are required to generate miRNAs and miRNA-mediated gene expression (Figure 1) (Ciafrè and Galardi, 2013). Immature miRNAs are normally translated into long primary transcripts (pri-miRNAs) with a stem-loop structure in the canonical miRNA biosynthesis pathway (Velázquez-Cruz et al., 2021). RBPs can influence the processing of pri-miRNA and precursor (pre-miRNA) biogenesis by identifying and binding sequences or special structures of the hairpin RNA. For example, RBP Lin28 can inhibit pri- and pre-let-7 miRNA biogenesis by interacting with the terminal loop of these immature miRNA *via* Drosha and Dicer (Mayr and Heinemann, 2013). Furthermore, overexpression of Lin28 was reported to increase glucose utilization in different tissues as well as prevent weight gain by suppressing let-7 miRNA biogenesis (Zhu et al., 2011; Shinoda et al., 2013). The interaction between Lin28 and let-7 miRNA may affect the pancreatic β-cell functions (Sung et al., 2019). RBPs have the ability to influence the stability and turnover of mature miRNAs (Ciafrè and Galardi, 2013; Connerty et al., 2015; Fukao et al., 2015). The Argonaute (AGO) proteins bind to double-stranded miRNAs and combine with them to form the miRNA-induced silencing complex (miRISC), in which one strand of the RNA duplex becomes functional while the other is deleted. The mature single-stranded miRNA generally binds to the 3′UTR of their target mRNAs, directing the translational inhibition and RNA degradation activity of miRISC. RBPs can bind to the 3′UTR of the target mRNAs to suppress the functions of miRNA *via* competing for the same binding motif or restructuring the target RNAs. In addition, RBPs can also alter the structure of 3′UTRs to facilitate miRNA binding, thereby promoting gene post-transcriptional regulation mediated by miRNA (Connerty et al., 2015; Velázquez-Cruz et al., 2021).

## 3 RNA-binding proteins and diabetes mellitus

### 3.1 Insulin secretion

Pancreatic islet β-cells are marked by their ability to synthesize and secrete large amounts of insulin, which maintain metabolic homeostasis *via* lowering glycemia (Campbell and Newgard, 2021). Although the pathogenesis of the two types of diabetes is not exactly consistent, T1DM and T2DM share common pathologies, such as decreased β-cell mass and loss of insulin secretory function (Eizirik et al., 2020). RBPs regulate a variety of processes in pancreatic β-cell, including insulin synthesis and secretion (Good and Stoffers, 2020; Demir et al., 2021). The abundant RNA-binding protein PTBP1 is the most well-studied regulator of insulin secretion (Magro and Solimena, 2013). In pancreatic β-cells, PTBP1 stabilizes preproinsulin mRNA by binding to the pyrimidine-rich region in its 3′UTR, thereby promoting the protein level of insulin. And it is regulated by glucose stimulation. In rat insulinoma INS-1 cells, suppression of PTBP1 by RNAi reduces insulin secretion (Knoch et al., 2004; Knoch et al., 2014). Besides, PTBP1 can also bind and stabilize 3′UTR of islet cell autoantigen (ICA512) mRNA, which is considered as an integral membrane protein of the insulin immature secretory granules (SGs). PTBP1 may stimulate the translation of insulin SG proteins *via* cap-independent mechanisms, which may be mediated by PTBP1 binding to the 5′UTR of the human preproinsulin (Ins2) mRNA (Knoch et al., 2004; Fred et al., 2011; Kulkarni et al., 2011; Knoch et al., 2014). In line with these finds, it was later discovered that the expression of PTBP1 in glucose-induced β-cells is mediated by the insulin receptor (IR) signaling pathway through Akt, and silencing Akt can significantly reduce the level of PTBP1 expression (Jeong et al., 2018). Accordingly, the level of RBP HuD decreased in β-cells of diabetes. HuD can bind to the Ins2 5′UTR to inhibit the translation of Ins2 and reduce insulin production. After glucose stimulation, Ins2 mRNA is promptly released from HuD, accompany by enabling translation of Ins2 mRNA. HuD knockout mice exhibit increased insulin levels in β-cells, while HUD overexpressed mice do the opposite (Lee et al., 2012). Furthermore, HuD enhances mitofusin 2 (Mfn2) expression level by binding to the 3′UTR of Mfn2 mRNA. In pancreatic β-cells, its decreased expression causes mitochondrial dysfunction (Hong et al., 2020). RBP hnRNPK, a member of the poly C-binding protein family, is phosphorylated and upregulated in islets under conditions associated with T2D. HnRNPK can bind to the poly C-rich fragments in JUND mRNA 3′UTR, thus influencing β-cell redox homeostasis and apoptosis. Post-transcriptional upregulation of JUND is blocked due to hnRNPK deletion during metabolic stress. Besides, DDX3X is essential for the efficient translation of JUND mRNA by interacting with hnRNPK (Good et al., 2019). In addition, overexpression of RBP Lin28a protects pancreatic β-cells from damage caused by streptozotocin (STZ) both *in vitro* and *in vivo*. Lin28a enhanced cell survival and proliferation through activating the PI3K-Akt signaling pathway, which is possibly regulated by let-7 (Sung et al., 2019). DDX1, an RNA-binding protein of the DEAD-box helicase superfamily, can promote translation activity of insulin mRNA by binding to its mRNA. Free Fatty Acids (FFAs) treatment causes DDX1 to be phosphorylated and dissociated from insulin mRNA, resulting in insulin translation inhibition (Li et al., 2018). RNA-binding protein CUGBP1 is upregulated in the islets of diabetic mice. CUGBP1 reduces insulin secretion in reply to glucose and GLP-1 stimulation by binding to the 3′UTR ATTTGTT sequence of PDE3B (Zhai et al., 2016).

RNA-binding proteins can not only directly bind to mRNA to regulate β-cell function and insulin secretion, but there have also been instances of RBPs and circular RNA interacting to exercise regulatory activities (Wu et al., 2022). For example, TDP-43 is a nuclear protein that acts as a regulator of gene expression as well as a DNA- and RNA-binding protein involved in RNA metabolism. It has recently been shown that knockout of TDP-43 in β-cells leads to defective insulin secretion (Araki et al., 2019). The intronic circRNA (ci-Ins2/ci-INS), generated by second intron excised from the primary insulin transcript, can interact with the RBP TDP-43 thereby controlling the expression of genes essential for insulin secretion in β-cells (Stoll et al., 2020). CircPPM1F competitively interacted with RBP HuR to suppress PPM1F translation, thus leading to pancreatic β-cell apoptosis through promoting M1 macrophage activation. Besides, two RBPs EIF4A3 and FUS might be oppositely regulated and maintained the expression of circPPM1F during the progression of T1DM (Zhang et al., 2020).

### 3.2 Insulin resistance

Tristetraprolin (TTP, also known as ZFP36) is an RBP that depresses post-transcriptional gene expression *via* interacting with AU-rich elements (AREs) in the 3′UTR of target mRNAs(Blackshear, 2002). TTP is induced by insulin stimulation *in vitro* and *in vivo* (Cao et al., 2008). The levels of TTP are decreased in the livers of diabetic mice and humans. TTP suppression may be due to insulin resistance and reduced AKT signal that regulates TTP at the promoter level under diabetic conditions. TTP binds to FGF21 mRNA 3′UTR leading to a degeneration of FGF21. TTP-KO mice may improve systemic glucose tolerance and insulin sensitivity by increasing liver-induced FGF21 (Sawicki et al., 2018). RBP hnRNP A1 interacts with glycogen synthase (gys1) and stabilizes its mRNA, thus facilitating glycogen synthesis in muscle tissue and preserving insulin sensitivity. Severe insulin resistance is caused by the absence of hnRNP A1 in mice fed a high-fat diet (HFD) (Zhao et al., 2020). In addition, it was also reported that myeloid-specific loss of TTP protects against glucose intolerance and improves insulin sensitivity in obesity (Caracciolo et al., 2018). Insulin-like growth factor binding protein (IGFBP1) modulates cellular responses independently of IGF binding through interaction of the Arg-Gly-Asp (RGD) sequence of IGFBP-1 with the cell surface integrin receptors. Previous studies have indicated that increasing the levels of circulating IGFBP1 improved insulin sensitivity in mice and humans (Gokulakrishnan et al., 2012; Rajwani et al., 2012). Furthermore, IGFBP1 increases insulin sensitivity by RGD integrin-binding domain and activation of focal adhesion kinase (FAK), thus improving glucose uptake in skeletal muscle cells. In response to glucose stimulation, RGD peptides can also increase insulin secretion of β-cells *via* FAK and integrin-linked kinase (ILK) activation (Haywood et al., 2017).

## 4 RBPs and diabetic complications

### 4.1 Diabetic neuropathy

Diabetic neuropathy affects at least half of patients with the development of diabetes. Patients with diabetes are characterized by signs of axonal degeneration and incomplete regeneration, demyelinating, and microangiopathy (Feldman et al., 2019; Calcutt, 2020). It has been reported that decrease of RNA-binding protein ZBP1 fails axonal RNA localization into the injured axons after sciatic nerve injury in T1DM rodent model induced by streptozotocin. This failure of RNA mobilization links to a reduction in axonal regeneration. When over-expression of ZBP1, this RBP can rescue *in vitro* growth defects in injured dorsal root ganglion (DRGs) from diabetic rats (Jones et al., 2021). Thus it shows that ZBP1 is a crucial savior in regeneration after axonal injury in diabetic rats.

*Elav-like* gene encodes Hu proteins, which belong to the RBPs superfamily. The Hu proteins family has three neuronal-specific members HuB, HuC, and HuD (encoded by *Elav-like 2,3,4* genes respectively), while the fourth is HuR or HuA (encoded by *Elav-like 1* gene) is omnipresent (Ambrosio et al., 2021). In another study, the neuronal-specific Hu proteins expression level was not correlated with its own gene in the thermal hypoalgesia condition caused by the advanced diabetic neuropathy. Moreover, the levels of *Elavl2* and *Elavl3* are reduced, while HuB is upregulated and HuD is downregulated in diabetic mice, compared to control one. Compared to control mice, *Elavl* genes and Hu proteins levels are significantly downregulated on the premise that algesic profile is unchanged under exposure to thermal radiation in diabetic-resistance mice (Mustăciosu et al., 2019). It has been certificated HuD protein upregulation in thermal hyperalgesia, which is the early phases of diabetes while otherwise in the thermal hypoalgesia condition caused by the advanced phases of diabetes (Sanna et al., 2014; Sanna et al., 2015). Previous studies indicated HuD can promote nerve regeneration and axon repair through interacting with mRNA by regulating its location or stabilizing the target mRNA (Wang et al., 2015; Gomes et al., 2017; Sanna et al., 2017). Therefore, it is reasonable to believe that the regulation of thermal hypoalgesia due to advanced diabetic neuropathy is closely related to changes in the post-transcriptional regulation of RNA in which RBPs are involved. What is more, the expression level of HuC in DRG neurons of rats with diabetic neuropathy is increased and is closely related to diabetic colonic hypersensitivity according to our unpublished research. We believe that the crucial role of Hu protein family in diabetic neuropathy can be further comprehended along with emerging research.

### 4.2 Diabetic nephropathy

Diabetic nephropathy (DN) is one of the most common chronic complications of both type 1 and 2 diabetes and is considered as a main cause of end-stage renal disease (ESRD). Glomerular basement membrane thickening, mesangial growth and hypertrophy, and the accumulation of extracellular matrix (ECM) proteins are all hallmarks of DN (Kanwar et al., 2011; Zoja et al., 2020). In a type 1 diabetes model, the RNA-binding protein HuR rapidly upregulated NAPDH oxidase 4 (NOX4) expression levels by binding to AU-rich elements (Ares) in the NOX4 mRNA 3′UTR, which induced mesangial cell (MC) fibrotic injury and kidney damage, and a reno-protective role was shown by suppressing HuR expression in type 1 diabetic mouse models (Shi et al., 2020). HuR can also bind to the 3′UTR Ares of the NOD2, increasing NOD2 expression and mRNA stability, which leads to glomerular mesangial cells damage and proteinuria in diabetic rats (Shang et al., 2015). In addition, transforming growth factor-β1 (TGF-β1) can cause mesangial extracellular matrix (ECM) proteins like collagen type 1-α2 (Col1a2) and type 4-α1 (Col4a1) to accumulate. Let-7 family miRNAs protect mouse mesangial cells (MMC) from collagen accumulation by inhibiting the levels of Col1a2 and Col4a1. Under diabetic conditions, elevated TGF-β1 expressions cause an increase in RBP Lin28b level, which is considered as a crucial inhibitor of let-7 miRNA biogenesis, thereby leading to the decrease of let-7 miRNA and the accumulation of mesangial ECM proteins (Park et al., 2014). RBP IMP2 can regulate the translation of Laminin-β2 (LAMB2), which is a component of the glomerular basement membrane and is associated with actin during translation. Decreased expression of IMP2 and Lamb2 in the diabetic condition leads to impaired mesangial cell migration and proteinuria (Schaeffer et al., 2012).

As noted above, there is a multitude of RBPs involved in the pathogenesis of glomerular mesangial cells damage and kidney injury. But do RBPs have an effect on renal parenchymal cells in diabetic conditions? The answer is obvious. Heterogeneous nuclear ribonucleoproteins (hnRNPs) are pre-mRNA binding proteins that can regulate the processing of mRNA. In renal proximal tubular cells of Akita hnRNP F-Tg mice, selective overexpression of RBP hnRNP F lowers expressions of angiotensinogen (Agt) and TGF-β1 and reduces kidney hypertrophy and glomerulotubular fibrosis (Lo et al., 2012). HnRNP F can suppress the transcriptional activity of rat Agt gene promoter by binding to the insulin-responsive element (IRE) (Wei et al., 2005). RNA-binding protein TTP expression was significantly reduced, while HuR expression was elevated in glomerular podocytes of patients with DKD and db/db mice. The expression of Interleukin (IL)-17 and claudin-1 are enhanced in the glomeruli, which are considered as targets of TTP and HuR. Treating db/db mice with GSK-3β small molecule inhibitors Eliminates changes in TTP and HuR in the glomeruli and mitigates overexpression of their target genes, which in turn also alleviates proteinuria and DKD pathology (Guo et al., 2020). It was known in previous studies that TTP may negatively regulate the progression of DKD, whereas HuR does the opposite (Khalaj et al., 2017; Ross et al., 2017). The imbalance between them may play a significant role in the occurrence and development of DKD. Moreover, glucose in high concentration could upregulate miR-138 level and repress the expression of SIRT1 by binding to its 3′UTR, resulting in the TTP inhibition in cultured podocytes as well as db/db mice renal tissues. Lower TTP expression causes an increase in the expression of inflammatory factors, leaving podocytes in an inflammatory state for an extended period of time, which leads to loss of normal morphology and function (Liu et al., 2021). RBP IGFBP-1 expression is reduced and affects the function of podocytes *via* β1-integrin/FAK signaling in human type 2 diabetic glomeruli (Lay et al., 2021).

### 4.3 Diabetic cardiomyopathy

Diabetic cardiomyopathy is a type of heart disease characterized by insulin resistance in heart tissue, compensatory hyperinsulinemia, and hyperglycemia progression which can give rise to heart failure (HF). And it occurs in the absence of basic cardiac diseases such as hypertension, coronary artery disease, and heart valve disease (Jia et al., 2018; Dillmann, 2019). CELF1, also known as CUG-BP, is a highly conserved RNA binding protein that regulates alternative splicing, polyadenylation, mRNA stability, and translation of target transcripts. Previous studies showed that CELF1 is up-regulating in the hearts of T1DM mice, but diabetes-induced AS alterations are consistent with CELF1 depletion or decreased CELF1 splicing activity (Blech-Hermoni et al., 2016; Belanger et al., 2018). Interestingly, RBFox2, an RNA-binding protein belonging to the RBFOX family, that is involved in AS regulation in heart diseases, shows the same trend as CELF1 (Gazzara et al., 2017; Verma et al., 2022). RBFox2 regulates cardiac function-related genes associated with diabetic cardiomyopathy. Though levels of RBFox2 protein are increased in the heart of diabetics, RBFox2 AS activity is low. This is due to the production of a dominant negative isoform of RBFox2 that blocks RBFox2-mediated AS, thereby damaging cardiomyocytes. Dominant negative RBFox2 expression is exclusive to diabetes and appears in its early stages, therefore it might be served as a potential target for treating diabetic cardiomyopathy (Nutter et al., 2016). Recently, research showed a spliced variant of RNA-binding protein PTBP1 is expressed aberrantly in T1DM mouse hearts compared with normal newborn mouse hearts. This PTBP1 spliced variant induced by diabetes has a lower inhibitory splicing activity. Furthermore, PTBP1 and RBFox2 regulate AS of some of their targets antagonistically (Belanger et al., 2019). Besides, another study indicated that CUG-BP (also known as CELF1)/RBFox2 can be phosphorylated and up-regulated by activating PKC signaling in diabetic heart, which in turn alters the AS of gene and contribute to diabetic cardiomyopathy pathogenesis (Verma et al., 2013). In addition, RBFox2 may regulate the AS of genes associated with cGMP-PKG-Ca^2+^ signaling pathway and lead to cardiomyopathy and heart failure (Wan et al., 2020). Lin28, an RNA-binding protein that comes in two forms: Lin28a and Lin28b, is essential for glucose metabolism (Zhu et al., 2011). Lin28a levels were significantly reduced in the diabetic mice hearts. Over-expression of Lin28a protects against diabetic cardiomyopathy through improving left ventricular ejection fraction (LVEF), promoting autophagy, and decreasing apoptosis, which is regulated by inhibiting activation of PKA/ROCK2 pathway (Sun et al., 2016). Moreover, Lin28a′s protective effects, induced by activation of autophagy, were dependent on Mst1 inhibition in diabetic mouse cardiomyocytes (You et al., 2020). Another RBP Quaking 5 (QKI) level was deficient in diabetic ob/ob mice myocardium. QKI-5 overexpression undermines the stability of FoxO1 mRNA thus inhibiting FoxO1 overactivation, which diminishes nitrosative stress and endoplasmic reticulum stress in ob/ob myocardium (Guo et al., 2014).

### 4.4 Diabetic cardiovascular disease

Vascular endothelial cell (EC) dysfunction is largely acknowledged as a major contribution to the pathophysiology of cardiovascular disease in people with diabetes. RBP QKI is a member of the signal transduction and activation of RNA (STAR) family, and it is linked to diabetic cardiomyopathy and atherosclerosis (Yang et al., 2018). Quaking 5 (QKI-5), Quaking 6 (QKI-6) and Quaking 7 (QKI-7) are three primary QKI transcript isoforms that have been reported to have important roles in the vascular system. For example, QKI-5 and QKI-6 have been demonstrated to play a key role in cardiovascular health regulation and maintenance through their involvement in a variety of processes such as EC and vascular smooth muscle cell differentiation, apoptosis, and neovascularization (Caines et al., 2019). Recently studies have implicated that QKI-7 expression in diabetic EC is elevated, and QKI-7 can bind to its downstream targets to promote their mRNA degradation. Furthermore, two RBPs CUG-BP and hnRNPM are involved in the regulation of QKI-7. It has been shown that these two RBPs are acting as a vital upstream factor of QKI-7 and regulating the transcription network in diabetes. An imbalance of CUG-BP/hnRNPM regulation causes up-regulation of QKI-7, which increases their target mRNA degradation and finally leads to diabetic endothelial dysfunction (Yang et al., 2020). Lin28 is an RBP involved in kidney and cardiac complications of diabetes (Park et al., 2014). Lin28 levels decreased in the hearts of T1DM mice (Sun et al., 2016; You et al., 2020). Emerging evidence has shown that Lin28 can prevent endothelial oxidative stress in response to high glucose by stabilizing OGG1 mRNA (Tao et al., 2021).

In addition to endothelial cell dysfunction, diabetic vascular disease can further alter capillary density to affect coronary flow velocity reserve (CFVR), which in turn develops into coronary microvascular disease (CMD) (Si et al., 2021). Previous research showed that HuR overexpression promotes angiogenesis *via* stabilizing VEGF-A mRNA and modifying endothelial cell angiogenic activity (Chang et al., 2013). In addition, diabetes attenuates the expression of Cx40, a gap junction channel protein, in cardiac ECs and impairs coronary microvascular function *via* downregulating the level of RNA-binding protein HuR. Overexpression of CX40 increased the density of capillary and ameliorated CFVR in diabetic mice (Si et al., 2021).

### 4.5 Diabetic retinopathy

Diabetic retinopathy (DR) is a common complication of DM, which remains a leading cause of vision damage or loss among working-age adults worldwide. Neovascularization plays an indispensable role in DR (Wang and Lo, 2018; Kang and Yang, 2020). DR involves early changes in the retina, characterized by vascular endothelial growth factor (VEGF) signal enhancement in various dysfunctions. It has been proven that inhibiting VEGF-mediated pathological angiogenesis enhances vision in DR patients (Stitt et al., 2013). Under the diabetic condition, the RNA-binding protein HuR is upregulated and binds to VEGF mRNA to regulate its stability, thereby enhancing its protein expression which leads to an abnormal increase in VEGF in the retina of diabetic rats (Amadio et al., 2010; Amadio et al., 2012). Besides, increased HuR and VEGF were suppressed by HuR silencing *via* intravitreal injection of small interfering RNA (siRNA) nanoparticles, which protect rat retinal tissue from damage caused by DM (Amadio et al., 2016). Furthermore, recent studies have shown that the expression of VEGF-A164 is time-specific (Bucolo et al., 2021). And VP12/14 and VP12/11, two derivatives containing indole structures, can regulate HuR expression and reduce the levels of VEGF and TNF-α release by human retinal endothelial cells (HRECs) exposed to high glucose (HG) conditions (Platania et al., 2020). HuR may represent a new target to inhibit the increased expression of VEGF, thus improving diabetic retinal vascular hyperplasia and inflammation. RNA-binding protein hnRNPA2B1 was confirmed to be a downstream target of Transthyretin (TTR) in human retinal microvascular endothelial cells (HRMECs). TTR can interact with hnRNPA2B1 to form a TTR-hnRNPA2B1 complex, which plays a critical role in TTR’s anti-angiogenesis function in hyperglycemia *via* the STAT4/miR-223e3p/FBXW7 signaling pathway (Gu et al., 2021). The expression level of RBP ZFR is meaningfully elevated both *in vitro* and *in vivo* in HRMECs in response to high glucose. Furthermore, ZFR can also enhance proliferation and migration in HRMECs. Besides, ZFR expression stimulated by high glucose can be attenuated by suppressing O-GlcNAcylation activity (Xing et al., 2019). The stable expression of RBP Lin-28 homolog b (lin28b) can promote VEGF expression (Wu et al., 2013; Weiße et al., 2020). It has been revealed that miR-152 can specifically target lin28b 3′UTR. Under high glucose conditions, miR-152 expression was significantly repressed, whereas lin28b expression was meaningfully augmented. Overexpression of lin28b increased the angiogenesis and the protein levels of proangiogenesis factors while inhibiting the function of miR-152 overexpression in both hRECs and hRMECs (Fu and Ou, 2020).

Furthermore, retinal neurodegeneration also occurs in the etiology of DR, which is mainly characterized by apoptosis and glial changes (Simo et al., 2018). Glia cells are considered as the interface between the vasculature and neurons (Hammes, 2018). mRNA Cap-binding protein eIF4E can recognize the structure of the 5′-m^7^GTP cap of mRNA and assemble it into eIF4F complexes, which can recruit ribosomes onto mRNA to perform translational functions. The recruitment of ribosomes, which can occur *via* a cap-dependent or cap-independent mechanism, limits the rate of mRNA translation. The interaction between 4E-BP1 and eIF4E promotes the dissociation of eIF4E from eIF4F complexes, which inhibits cap-dependent and promotes cap-independent translation (Schrufer et al., 2010; Dennis et al., 2015; Miller et al., 2016). In Muller cells and retina of diabetic rats, high glucose conditions increase REDD1 levels and enhance the binding of 4E-BP1 to eIF4E. This reduces the overall rate of protein synthesis and cap-dependent mRNA translation accompanied by upregulated cap-independent VEGF mRNA translation, which is thought to be a key mechanism in the development of DR (Dennis et al., 2015). In addition, retinal protein O-GlcNAcylation promotes cap-independent Cd40 mRNA translation through a 4E-BP1 dependent mechanism under a diabetic condition in Muller glia cells. Elevated expression of the CD40 protein in Muller glial cells leads to chronic retinal inflammation correlates with DR (Dierschke et al., 2020).

## 5 Conclusion and prospects

**FIGURE 2:**
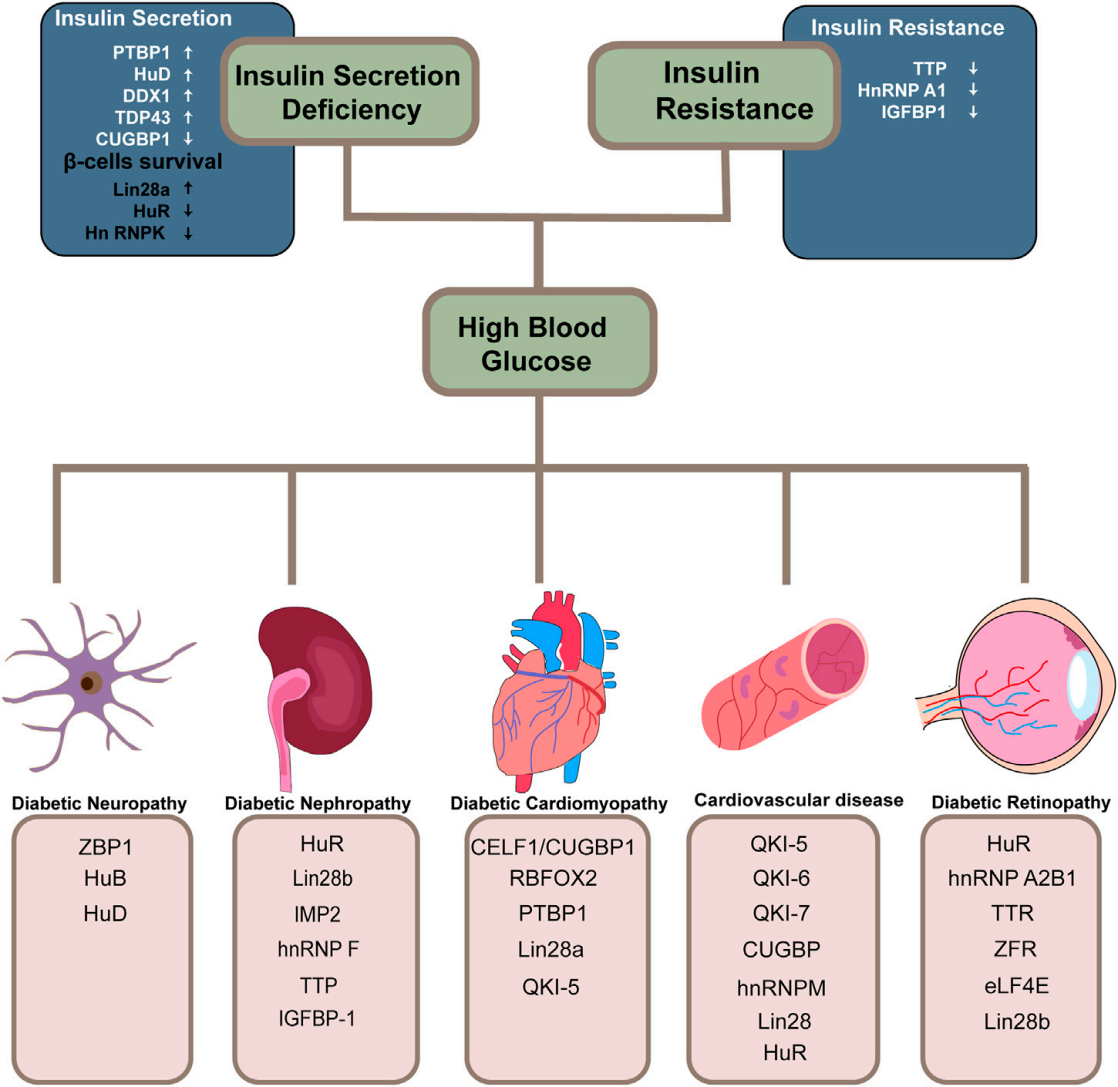
Overview of RBPs involved in diabetes and its related systematic complications. RNA-binding proteins implicated in the two decisive links in the progression of diabetes and its related systematic complications are summarized. The arrows in the diagram point to either protective effects or the opposite.

Emerging evidence indicates that dysregulation of RBPs is linked to a variety of disorders and affects almost every stage of the disease progression. There is a lot of literature on RBPs and their implicates in diabetes, but it is fragmented and lacks systematic reviews. With a high incidence of diabetes and severe chronic complications of multiple systems, a thorough understanding of the post-transcriptional regulatory role of RBPs in diabetes and its complications is critical to the development of novel RNA-based therapies. Here, for the first time, we categorize and summarize some common and relatively mature RBPs according to different systemic complications (Figure 2) (Table 1). We hope that both new therapeutic developers and researchers working on RBPs in the diabetes field can find some convenient and useful information from this review.

**TABLE 1:** The regulatory mechanism of RNA-Binding proteins and the outcomes of their dysregulation in diabetic complications.

RBPs	Diabetic complications	Post-transcriptional mechanisms involved in diabetic complications	Outcomes associated with RBPs dysregulation in diabetic complications
ZBP1	Diabetic neuropathy	mRNA location	Reduce injured axon regeneration
HuD	Diabetic neuropathy	mRNA location/stability	Promote nerve regeneration
Lin28b	Diabetic nephropathy	MiRNA biogenesis	Promote mesangial extracellular matrix proteins accumulation by inhibiting let-7 miRNA biogenesis
	Diabetic retinopathy	mRNA translation	Suppress angiogenesis in hRECs and hRMECs
IMP2	Diabetic nephropathy	RNA translation	Promote mesangial cell migration by regulating the translation of LAMB2
hnRNPs	Diabetic nephropathy	RNA translation	Over-expression in RPTCs can attenuate systemic hypertension and kidney hypertrophy
hnRNPA2B1	Diabetic retinopathy	MiRNA activity inhibition	repress neovascularization in DR
HuR	Diabetic nephropathy	mRNA translation	Mesangial cell fibrotic injury and kidney damage
	Diabetic cardiovascular disease	mRNA stability	Modify endothelial cell angiogenic activity
	Diabetic retinopathy	Post-transcriptional modifications	Improve the expression level of VEGF and cause diabetic retinal vascular hyperplasia and inflammation
TTP	Diabetic nephropathy	mRNA degradation	The imbalance between TTP and HuR promotes podocyte injury and inflammation in DKD
CELF1 (CUG-BP)	Diabetic cardiomyopathy	Alternative splicing	Low splicing activity and activate PKC signaling in diabetic hearts
RBfox2	Diabetic cardiomyopathy	Alternative splicing	Low splicing Activity; lead to the development of cardiomyopathy and heart failure
PTBP1	Diabetic cardiomyopathy	Alternative splicing	Low inhibitory splicing Activity; PTBP1 and RBfox2 regulate splicing antagonistically
Lin28a	Diabetic cardiomyopathy	RNA translation	Over-expression can promote LVEF, autophagy and decrease apoptosis
QKI5	Diabetic cardiomyopathy	mRNA stability	Over-expression can diminish nitrosative stress and endoplasmic reticulum
QKI7	Diabetic cardiovascular disease	mRNA degradation	Diabetic endothelial Dysfunction
Lin28	Diabetic cardiovascular disease	mRNA stability	Prevent endothelial from oxidative stress by stabilizing OGG1 mRNA
ZFR	Diabetic retinopathy	Post-translational modifications	aggravate proliferation and migration induced by high glucose in HRMECs
eLF4E	Diabetic retinopathy	mRNA translation	Chronic retinal inflammation

In this review, we focus on the molecular mechanisms of RBPs and mRNA interactions, which have an either positive or negative impact on diabetes. The functional interactions between RBPs and non-coding RNAs, including microRNAs and circular RNAs, are another essential aspect that is briefly explored in this study. We have spent a lot of sections discussing that dysregulation of RBP leads to abnormal function of its interacting nucleic acids or proteins in diabetes. However, RBP’s own activity is profoundly controlled by post-translational modifications (PTMs), Which is also an important mechanism that determines the occurrence and development of diseases (Lovci et al., 2016). PTMs generally refers to enzymatic reactions that occur after protein synthesis. PTMs follow a variety of signaling transductions that induce proteins to form covalent bonds with new functional chemical groups such as phosphate, methyl, acetyl, and ubiquitin (Deribe et al., 2010). PTMs can significantly alter the activity and properties of RBPs, resulting in changes in regulating protein activity, stability, localization, turnover and degradation (Velázquez-Cruz et al., 2021). Phosphorylation is the most common and widely explored among various types of PTMs. For example, several specific phosphorylations of hnRNPK by specific kinases can alter hnRNPK protein subcellular localization, stability, or affinity for binding targets (Xu et al., 2019). PTMs can also alter the Subcellular localization of HuR, and most phosphorylation of HuR occurs in its hinged region (Grammatikakis et al., 2017). In addition, the nuclear import of serine/arginine-rich (SR) protein family requires phosphorylation by the SR protein kinases 1 and 2 (SRPK1/2) (Long et al., 2019). These post-translational modifications also play an important role in diabetes. O-linked N-acetylglucosamine (O-GlcNAc) glycosylation is involved in the pathogenesis of diabetes and its related complications by O-linked addition of GlcNAc (O-GlcNAcylation) to Ser/Thr residues of proteins (Issad et al., 2010; Zhu and Hart, 2021). There are multiple studies reported that O-GlcN acylation enhancement of retinal proteins in rodent models of type 1 and type 2 diabetes (Mellor et al., 2015; Peterson and Hart, 2016; Masaki et al., 2020). The cap-binding protein eIF4E is more readily sequestered in the mice with DR, due to the repressor of mRNA translation 4E-BP1 being O-GlcNAcylated (Dierschke et al., 2019). O-GlcNAc signaling activation also increases the level of RBP ZFR under high glucose condition, which aggravates proliferation and migration induced in HRMECs (Xing et al., 2019). It is not difficult to grasp that PTM as a major element governs the properties and function of RBPs with highly dynamic and largely reversible. We do not describe in detail the regulation of PTMs to RBPs and the complex signaling pathways it orchestrates. However, a thorough understanding of the molecular underpinnings of disease-associated PTMs dysregulation on RBPs is necessary for fully comprehending the pathophysiological process of diseases.

In summary, we expect to fully understand the dynamic RBPs-mediated regulatory network in diabetes. Correcting gene expression abnormalities in diabetic patients by targeting the interaction between RBPs and their target RNAs could be an effective approach. RNA-based therapies are primarily designed drugs to imitate or antagonize specific RNA processes by mimicking the action of protective RBPs or inhibiting the action of pathogenic RBPs. Many candidate strategies are being applied to target RBPs for therapeutics in pre-clinical or clinical trials, such as small-molecule inhibitors, therapeutic small peptides, anti-sense oligonucleotides (ASOs), and siRNA (Chi et al., 2017; Mohibi et al., 2019). Besides, circular RNAs are also considered as a potential strategy that can be designed to bind RBDs of RBPs and compete with target RNAs (Mohibi et al., 2019). However, how to improve the target specificity is also a tough problem that needs to be overcome. Although many issues and connections of RBPs remains to be explored and solved, existing knowledge and growing evidence show that we have an opportunity to enter a new era in the therapies of diabetes and its related complications.
